# Risk for Eating Disorders Modulates Startle-Responses to Body Words

**DOI:** 10.1371/journal.pone.0053667

**Published:** 2013-01-16

**Authors:** Cornelia Herbert, Andrea Kübler, Claus Vögele

**Affiliations:** 1 Department of Psychology I, University of Würzburg, Würzburg, Germany; 2 Performance Psychology, Institute of Psychology, German Sport University Cologne, Cologne, Germany; 3 Research Unit INSIDE, University of Luxembourg, Luxembourg City, Luxembourg; University of Ulm, Germany

## Abstract

Body image disturbances are core symptoms of eating disorders (EDs). Recent evidence suggests that changes in body image may occur prior to ED onset and are not restricted to in-vivo exposure (e.g. mirror image), but also evident during presentation of abstract cues such as body shape and weight-related words. In the present study startle modulation, heart rate and subjective evaluations were examined during reading of body words and neutral words in 41 student female volunteers screened for risk of EDs. The aim was to determine if responses to body words are attributable to a general negativity bias regardless of ED risk or if activated, ED relevant negative body schemas facilitate priming of defensive responses. Heart rate and word ratings differed between body words and neutral words in the whole female sample, supporting a general processing bias for body weight and shape-related concepts in young women regardless of ED risk. Startle modulation was specifically related to eating disorder symptoms, as was indicated by significant positive correlations with self-reported body dissatisfaction. These results emphasize the relevance of examining body schema representations as a function of ED risk across different levels of responding. Peripheral-physiological measures such as the startle reflex could possibly be used as predictors of females’ risk for developing EDs in the future.

## Introduction

What do you see and feel when you look in the mirror? When asked this question more than 80% percent of women in North America and almost 44% of women in Europe report to be dissatisfied with their physical appearance expressing feelings of being inappropriate, fat, ugly and afraid of their body even though self-perception as being overweight might be unjustified [1], [2].

Body image disturbances constitute important risk factors for eating disorders (EDs), [3] and are key diagnostic features in Anorexia Nervosa (AN) and Bulimia Nervosa (BN) (DSM-IV). Theoretically, the term body image refers to a person's perception of his or her own physical appearance, i.e. how they perceive themselves when looking into a mirror, or when picturing themselves in their mind in relation to other people. Body image is defined as a multi-dimensional concept that includes perceptual and cognitive-affective and behavioral components [4].

Body image disturbances in EDs have been studied at a subjective-behavioral level and with regard to underlying neural mechanism [5], [6], for a meta-analytic review see [3], [7]. Results converge on profound biases in all three body image components in women diagnosed with AN or BN compared to women without EDs. Women with AN and BN constantly overestimate their own body size in tests based on perceptual judgments, they show marked negative attitudes towards their own body, and demonstrate increased avoidance behavior when directly confronted with their own body. Studies using neurophysiological methods such as functional magnetic resonance imaging (fMRI) found that during confrontation with pictures of their own body or of bodies assessed as intolerable women diagnosed with AN and BN demonstrate altered activity in brain regions that form part of the brain’s fear (amygdala) and emotion control networks (e.g. medial prefrontal cortex, fronto-parietal cortex) [8]–[12]. In individuals with AN, brain activity patterns elicited in response to exposure to the own body often parallel those typically seen in patients with anxiety disorders when confronted with threat relevant material [13].

In a more recent functional imaging study [14], women with AN showed enhanced activity in the amygdala, whereas those with BN responded with enhanced activity in the medial prefrontal cortex even when confronted with more abstract cues such as body shape and weight related words. Importantly, in a study by Shirao et al. [15] changes in emotional brain circuits in response to body words were found in women without a history and current diagnosis of an ED, but who were at risk for EDs as defined by severity of psychological and behavioral ED symptoms on the Eating Disorder Inventory. Participants were asked to process a series of body words while their brain activity was scanned by means of fMRI. Reading of body words lead to activity changes in the amygdala and in parahippocampal brain regions involved in autobiographical memory retrieval [16], while these activity changes were correlated with participants’ scores on the Eating Disorder Inventory, see also [17].

The results of the latter two studies using verbal and thus more symbolic body related cues are of particular theoretical interest. Their findings support the notion that alterations in body image perception in EDs are not limited to in-vivo exposure to one’s own body image or morphed images thereof, but extend to difficulties in correctly representing body schemata in memory [18]–[20]. Importantly, these ‘mental-representational’ changes might well exist prior to ED onset and thus be considered risk factors for the development of an ED.

If these changes in the mental representation of body related information exist prior to onset of an ED, it is theoretically and clinically important to determine whether they are only evident at a cortical (e.g. as altered brain activity patterns) and subjectively experiential level (e.g. subjective ratings and evaluation of body related stimuli), or if they are already of motivational and behavioral relevance. In the latter case, exposure to body related words in women at risk for EDs should be accompanied by changes in peripheral-physiological responses such as startle or heart rate reactivity.

Changes in startle and heart rate during processing of salient stimuli indicate the extent of motivational engagement and the priming of approach and avoidance tendencies which regulate approach and avoidance behavior in response to stimuli that protect or challenge individual well-being [21]. When confronted with emotionally negative, unpleasant stimuli amplitudes of the startle eye blink response are potentiated, both in relation to stimuli of pleasant or neutral content and inhibited when confronted with emotionally positive, pleasant stimuli. This modulation of the startle response is typically more pronounced for highly arousing emotional stimuli and has been replicated many times by probing the startle response during viewing of emotional stimuli such as pictures [22] and recently also with words [23], [24].

Cardiovascular reactivity to emotional stimuli has also been investigated. Previous studies have mostly used visual stimuli of different affective values and found a marked cardiac deceleration when viewing unpleasant pictures [25]. This initial heart rate deceleration is thought to reflect enhanced orienting toward stimuli of emotional relevance [26], [27]. In eating disorders both measures (startle reflex and heart rate) have been successfully used to determine an individual’s spontaneous reactions toward food stimuli or pictures of female bodies [28]–[30].

In the present study startle modulation and heart rate reactivity were used in addition to subjective measures to examine the extent of motivational engagement in the processing of body words in young women with no history of EDs. We aimed at investigating whether healthy women would present with a negativity bias toward body related concepts, whether this bias is attributable to a general negativity bias in the processing of body related information or specific to women at risk for EDs. Provided specific, we hypothesized to find increased defensive physiological reactions during confrontation with body related concepts in women at risk for EDs. In particular, we were interested which of the diagnostically relevant risk factors would drive these effects (drive for thinness, bulimia or body dissatisfaction).

## Materials and Methods

### Ethics Statement

The experiment was conducted in accordance with the Declaration of Helsinki (World Medical Association). All participants gave written informed consent prior to participation. The study design and methodology using words and peripheral-physiological methods such as startle in healthy samples including information regarding informed consent were approved by the ethical committee of the German Psychological Society (http://www.dgps.de/en/).

### Participants

Forty-one females, all native speakers of German with comparable social background (high school degree as lowest qualification) were recruited via advertisements at the University of Würzburg. Startle and heart rate data of four participants had to be excluded from analysis due to too much noise in ECG (N = 3) or EMG (N = 1) signals. Data of two further individuals had to be excluded because of missing ratings or questionnaire data. Exclusion of these subjects did not introduce a systematic bias with regard to ED risk, mood or BMI. The final sample comprised 35 females (mean age: 22.9, SD = 2.3).

Prior to experimental testing participants were asked to provide information on their health (custom made questionnaire to assess drug use, chronic physical conditions, neurological diseases, mental ill health and eating-, exercise-, and smoking habits). None of them reported to smoke more than 5 cigarettes a day, none of them was under medication affecting the brain or heart. In this context subjects also reported when they had their last meal, snack, caffeine drink or cigarette and rated their bodily sensations on Likert scales to control for craving, hunger or feelings of satiation.

Height and weight were assessed by self-report to calculate the body mass index (BMI). For the assessment of current mood, depression and anxiety participants filled in the Positive Affect Negative Affect Schedule (PANAS) [31], the Beck Depression Inventory (BDI) [32] and the Spielberger Anxiety Inventory (STAI) [33].

Risk for EDs and symptoms frequently related with EDs were assessed with the Eating Disorder Inventory (EDI) [34]. The EDI asks for characteristic symptoms related to EDs (AN and BN, in particular). It consists of eight scales including *drive for thinness* (excessive concern with dieting and preoccupation with weight due to fear of gaining weight), *bulimia* (episodes of binge eating and purging), *body dissatisfaction* (strong dissatisfaction with one's own physical appearance including continuing negative thoughts and feelings about one’s own body), *ineffectiveness* (feelings of inadequacy, insecurity, and worthlessness), *perfectionism* (not being satisfied with anything less than perfect), *interpersonal distrust* (reluctance to form close relationships), *interoceptive awareness* (the ability to discriminate between sensations and feelings including hunger and satiety), and *maturity fears* (fear of facing the demands of adult life). The first three scales drive for thinness, bulimia and body dissatisfaction describe diagnostically relevant symptoms of anorexia nervosa and bulimia nervosa and in line with the present study have been used in previous research to identify individuals at risk for EDs in nonclinical samples [15], [17], [34], [35].

Table 1 summarizes results of self-report data for the current sample. Females scored within the normal range on the BDI, the STAI state and trait anxiety scales, and reported more positive than negative mood. Body mass index (kg/m^2^) ranged from 17.04 to 25.5 (mean: 21.3, SD = 2.16). On average, EDI scores were comparable with norms reported in female samples without a diagnosis of ED [35]. Scores on the three diagnostically relevant EDI subscales drive for thinness, bulimia and body dissatisfaction ranged from 8–38 (mean: 16.51, SD = 7.6), 7–30 (mean: 12.06, SD = 4.95) and 13–50 (mean: 28.7, SD = 10.53). Nine women reported high drive for thinness (scores >20.5); ten had high bulima scores (scores ≥14), and ten were extremely dissatisfied with their own body (scores ≥37; i.e. scores greater than the upper 25 percentile of the studied sample). Few women reported extremely low drive for thinness (N = 8, scores <11), bulimic symptoms (N = 6, scores <9) or body dissatisfaction (N = 9, scores <19.5, i.e. scores lower than the first 25 percentile).

**Table 1:** Self-report data (questionnaires).

N = 35 females	Mean (SD)
EDI_Drive for Thinness	16.51 (7.6)
EDI_Bulimia	12.06 (4.95)
EDI_Body Dissatisfaction	28.7 (10.53)
BSQ_Body Shape Concerns	74.06 (30.62)
BCQ_Body Checking Behaviour	42.3 (11.14)
SAAS_Social Appearance Anxiety	31.8 (12.2)
STAI_State Anxiety	37.81 (8.4)
STAI_Trait Anxiety	37.49 (9.76)
BDI_Depression	5.40 (5.1)
PANAS_Negative Affect	13.34 (4.2)
PANAS_Positive Affect	27.97 (6.0)
BMI_Body Mass Index (kg/m^2^)	21.3 (2.1)
Age (years)	22.9 (2.3)

### Procedure

The study was conducted in one of the Psychophysiology Laboratories of the Department of Psychology I at the University of Würzburg. Upon arrival participants were informed about the study and signed informed consent. Next, they were seated in a comfortable chair, and electrodes were attached for the assessment of startle responses and heart rate recordings. Then the participants were asked to fill in the health questionnaire, followed by a description of the experimental procedure in general terms. Participants were told that for the next 20 minutes they would be presented with a series of words displayed in the centre of a computer monitor. They were asked to read each word silently and to attend to the word until a fixation cross appeared on the screen. In addition, participants were told that a sudden loud noise burst would occasionally be heard biaurally via stereo headphones, which they should ignore. Immediately - and unexpectedly - after the experiment, they were asked to recall as many of the words as possible (free recall test), and then to rate the presented words for perceived valence and arousal on the Self-Assessment Manikin Scales [36]. Finally, participants filled in the EDI before they were debriefed and thanked for participation including financial reimbursement.

### Stimulus Material and Task

Experimental stimuli comprised 33 body related words and 33 neutral words (e.g. chair, desk, printer etc.). Body words described mainly body parts and body weight related concerns associated with these parts (e.g. hips, thighs, arms, legs, roll of flab, etc.). Body words were taken from a word corpus created by our own research group. The corpus comprises a total of 86 nouns, which were rated for valence and arousal with the Self Assessment Manikin by a separate, normative sample of 60 women (mean age: 23.5 years, SD = 5.6 years) with a social background and age comparable to the current sample. Body words receiving high negative valence and arousal scores in the normative female sample were chosen and matched for word length and word frequency with neutral nouns. Neutral nouns were selected from German word lists [37], [38], which provide normative ratings of valence and arousal for German words. Only nouns that received low arousal and moderate valence scores were chosen for the neutral word sample. Arousal and valence differed significantly between body and neutral words. Word length and frequency did not.

Body words and neutral words were presented in randomized order in black font (font Times; size = 40) on white background in the centre of a 19-inch computer monitor. Words were shown for 5 seconds and followed by a fixation cross that stayed for 6 seconds in the middle of the screen. Startle tones (95 dB sound pressure level burst of white noise, 50 ms duration, instantaneous rise and fall times) were presented acoustically during viewing of body related and neutral words via stereo headphones. Startle probes occurred in two third of the trials (e.g. 22 body and 22 neutral words were paired with startle) in the time window from 1–3.5 seconds after word onset, which accords with the literature investigating affective modulation of the startle reflex with pictures or words [23], [24], [39].

Experimental runs were controlled by Presentation software (Neurobehavioural Systems Inc.).

### Physiological Recordings

#### Startle eye blink and heart rate

The startle eye blink response was measured electromyographically with two miniature Ag/Ag electrodes (5 mm diameter) attached to the m. orbicularis oculi beneath the left eye. Inter-electrode distance between the two electrodes was kept constant across participants. Recording and analysis of startle data was performed according to the standard guidelines for startle eye blink measurement [40]. Heart rate was measured via the electrocardiogram (ECG) using the Eindhoven lead I configuration for which two Ag/AgCl electrodes (5 mm diameter) were attached to the left and right wrists.

Electromyographic and electrocardiographic signals were sampled continuously at 2500 Hz from DC to 1000 Hz. Signals were measured via bipolar channels using BrainAmp amplifier (BrainProducts GmBH) and BrainVision Recorder software (BrainProducts GmBH). Impedance was kept below 5 kOhm for both biosignals; an electrode placed at the participant’s neck was used as ground.

Electromyographic and electrocardiographic signals were preprocessed and analyzed offline with BrainVision Analyzer 2 software (BrainProducts GmBH). Raw EMG signals were filtered offline from 28–500 Hz, rectified, and baseline corrected using the 100 ms pre-stimulus period for baseline correction. For each startled word, startle peak amplitude was analyzed from the filtered, rectified and baseline corrected EMG signal, which was additionally smoothed by a moving average for peak amplitude detection. EMG peak amplitude (microvolt) was computed for each trial and averaged separately for body words and neutral words. Data was inspected for outliers, trials with a response onset 20 milliseconds before tone onset and peak latency greater than 180 ms after tone onset were excluded from analysis as were trials where no clear peak amplitude could be detected.

Raw ECG signals were filtered offline from 1–40 Hz and visually inspected for movement artifacts and ectopic beats. Electrocardiographic signals were then segmented from 1000 ms prior to stimulus onset until 5 seconds after stimulus-offset and baseline corrected using the 1000 msec pre-stimulus period. R-peaks were detected by a semi-automated peak detection algorithm. R-R intervals were calculated within the time window from 0–10 seconds post stimulus onset for each trial. R-R intervals (Inter-beat-intervals, IBIs) were then converted to heart rate (HR) in beats-per-minute (bpm). Mean heart rate and changes in heart rate during reading of body words and neutral words were analyzed from 0–10 seconds post stimulus onset. To exclude startle induced changes in phasic heart rate, mean heart rate as well as changes in heart rate were analyzed for non-startle trials only.

### Analysis

#### Startle eye blink and heart rate

Startle modulation was statistically analyzed with a repeated measures analysis of variance (ANOVA) including the factor *word category* (body vs. neutral) as within-subject factor. Mean heart rate and changes in heart rate during reading of body words and neutral words were analyzed with repeated ANOVAs including the factors *word category* (body vs. neutral) and *time* (1 second intervals from 0–10 seconds after word presentation) as within-subject factors. Heart rate change scores were calculated to determine the maximal change in HR observed during reading relative to HR at word onset. Change scores were calculated for body words and neutral words and compared with repeated ANOVAs with *word category* (body vs. neutral) as within-subject factor. To evaluate changes in startle, heart rate and subjective evaluations as a function of females’ risk for EDs, groups were formed for the three diagnostically validated subscales of the EDI Inventory, i.e. drive for thinness (N = 23 vs. N = 12, median score: 16), bulimia (N = 21 vs. N = 14, median score: 11) and dissatisfaction with one’s own body (N = 18 vs. N = 17, median score: 27) by using median split. Subjects with exactly the median score were included into the low ED risk groups. This was particularly the case for bulimia (N = 7) and drive for thinness (N = 6) and to a lesser degree for body dissatisfaction (N = 2). Separate ANOVAs were then re-calculated with the respective factor *group* (low vs. high drive for thinness, bulimia, or dissatisfaction with one’s own body) as between-subject factor. Significant main and interaction effects were decomposed by planned comparison tests. In addition, correlation analyses (Pearson, two-tailed) were performed to further determine the relationship between the risk for EDs and changes in startle and heart rate during exposure to body and neutral words. Startle difference scores (subtracting for each participant startle amplitude of neutral words from those of body words), and absolute HR and HR change scores were used for correlation analyses. To further control for possible relationships between females’ risk for ED and differences in affective variables such as depression, anxiety or current positive and negative affect, scores obtained from mood questionnaires were also correlated with startle and heart rate data.

#### Subjective ratings and free recall

Participants’ ratings (valence, arousal) and their memory performance in the free recall task were analyzed with repeated ANOVAs, each including the within-subject factor *word category* (body vs. neutral) and the between-subject factor *group* (low vs. high drive for thinness, or bulimia or dissatisfaction with the own body). Ratings and memory data were correlated with participants’ questionnaire data.

## Results

### Startle Eye Blink

**Figure 1:**
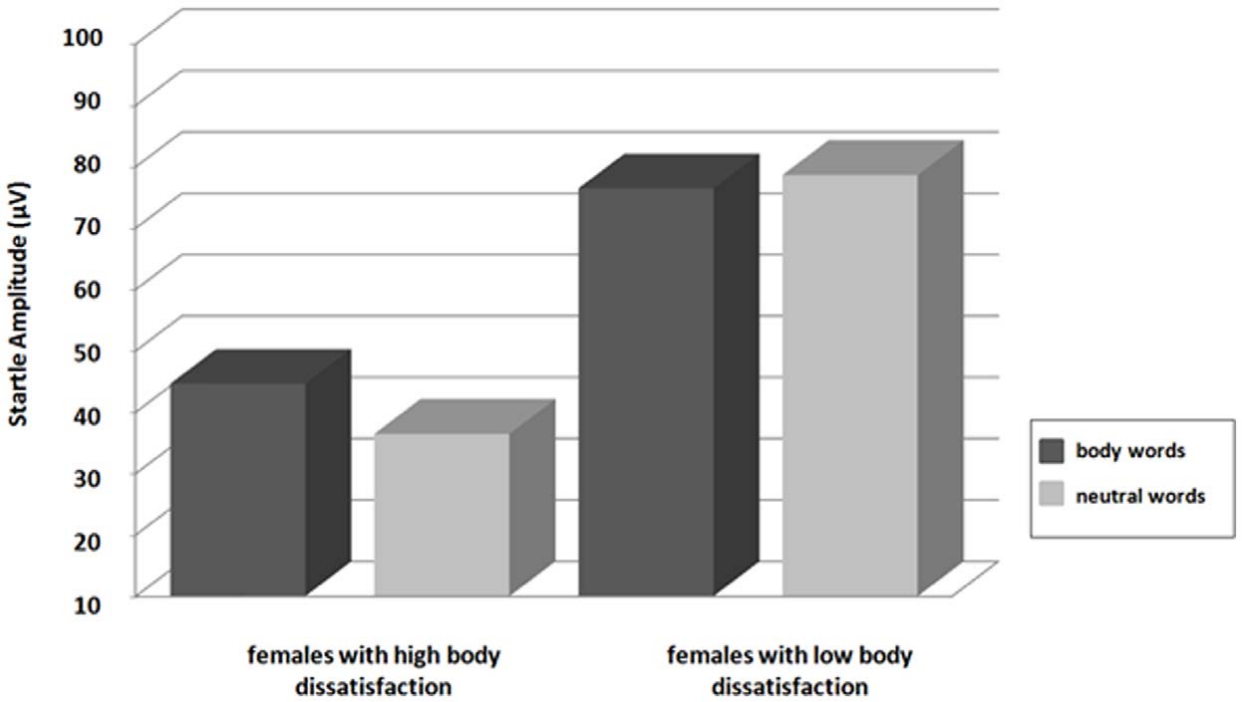
Startle modulation during reading of body words and neutral words in females reporting high (N = 17) vs. low (N = 18) body dissatisfaction on the EDI subscale body dissatisfaction.

Startle modulation did not differ significantly during reading of body words and neutral words, *F*(1,34) = 0.36, p>.05. However, a significant interaction between *word category* and *group* was observed for the EDI subscale body dissatisfaction, *F*(1,33) = 4.5, p<.05, suggesting larger startle amplitudes for body words compared to neutral words in women reporting high body dissatisfaction (Figure 1). The factor *group* was not significant, *F*(1,33) = 3.7, p>.05. No significant interactions between *word category* and *group* were observed for drive for thinness, *F*(1,33) = 1.6, p>.05, and bulimia, *F*(1,33) = 2.2, p>.05.

Correlation analyses revealed positive relationships between startle difference scores and the EDI subscale body dissatisfaction (r = .337, p<.05). There were no significant correlations with drive for thinness (r = .144, p>.05) or bulimia (r = .268, p>.05). Difference scores did further not correlate significantly with depression scores (r = .126, p>.05), current positive (r = −.174, p>.05) or negative affect (r = .089, p>.05), or trait and state anxiety (state anxiety: r = .126, p>.05; trait anxiety: r = .186, p>.05). In addition, there was no significant relationship with body weight (BMI: r = .188, p>.05), suggesting that correlations between startle and ED symptoms were not mediated by inter-individual differences in affect or body mass.

### Heart Rate

**Figure 2:**
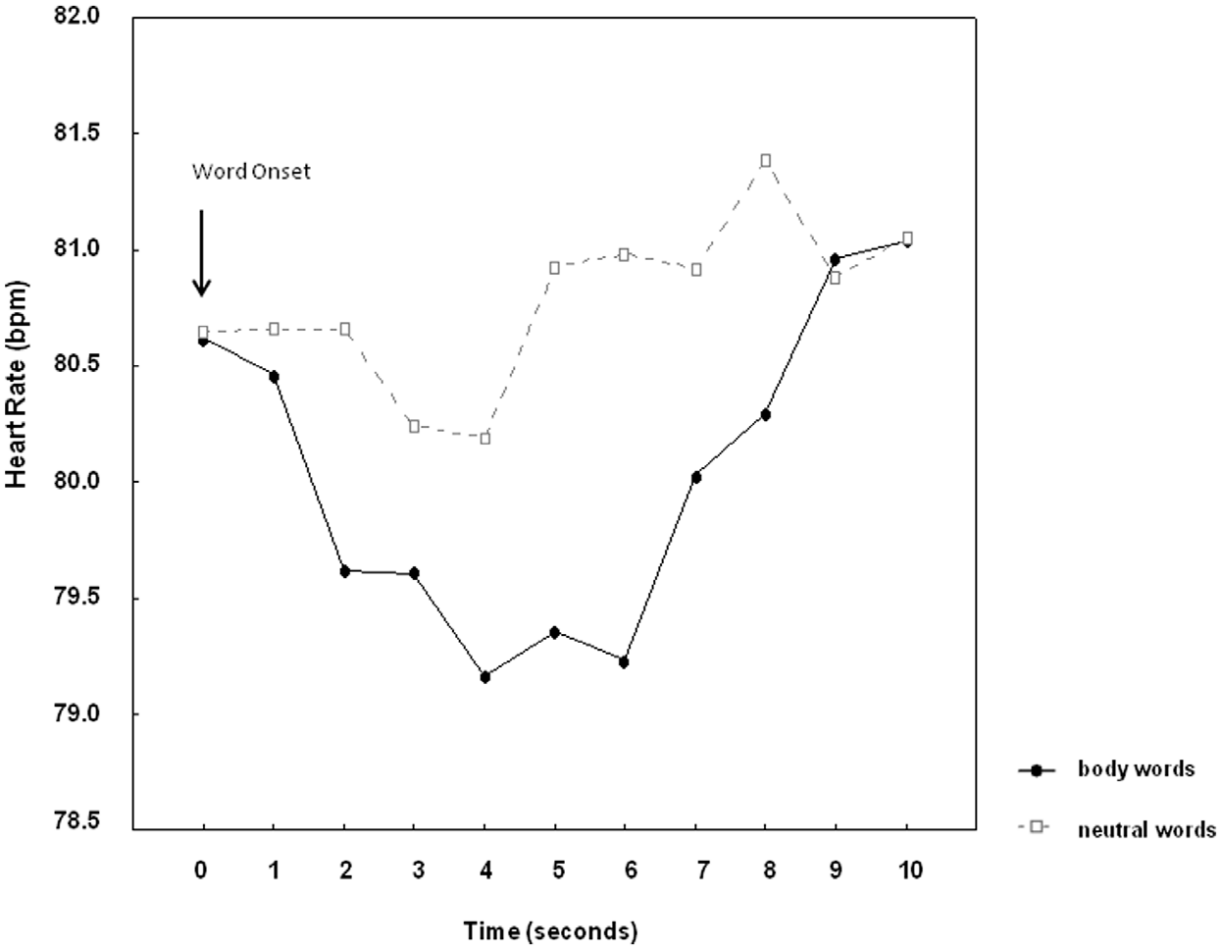
Changes in heart rate during reading of body words and neutral words from 0–5 seconds after word onset. The time interval from 5–10 seconds represents the interstimulus interval following word offset.

Mean HR tended to be lower during reading of body words (M = 79.9, SEM = 2.7) compared to neutral words (M = 80.7, SEM = 2.6), *word category, F*(1,34) = 3.01, p = .09. Word reading elicited a prominent heart rate deceleration, *time, F*(9,306) = 2.12, p<.05. During reading, heart rate deceleration was most pronounced in the time window from 2–6 seconds after word onset (Figure 2). Maximal heart rate deceleration in this time window was significantly greater for body words than for neutral words, *F*(1,34) = 5.37, p<.05. No main effects of *group* and no interactions between *word category* and *group* were observed, neither for body dissatisfaction, nor for bulimia or drive for thinness. Neither heart rate nor heart rate deceleration were correlated to measures of ED risk, BMI or mood.

### Subjective Ratings and Free Recall

Body words were rated more negative in valence and higher in arousal compared to neutral words, category_valence:_ *F*(1,34) = 47.1, p<.001, category_arousal:_ *F*(1,34) = 37.1, p<.001. Interactions between the factors *word category* and *group* revealed significantly greater differences in valence and arousal ratings between body words and neutral words in women reporting high vs. low body dissatisfaction (p<.001), drive for thinness (p<.001), or bulimic symptoms (p<.05). Mean valence and arousal ratings are summarized in Table 2.

**Table 2:** Valence and arousal ratings of body words and neutral words in females scoring high or low on the three diagnostically relevant subscales of the Eating Disorder Inventory.

	Body	Neutral
***High Body Dissatisfaction***		
Valence	4.2 (0.64)	5.3 (0.42)
Arousal	3.8 (1.03)	2.21 (1.09)
***Low Body Dissatisfaction***		
Valence	4.9 (0.82)	5.4 (0.71)
Arousal	3.3 (1.37)	2.83 (1.42)
***High Bulimia***		
*Valence*	4.2 (0.67)	5.3 (0.39)
*Arousal*	3.5 (1.23)	2.04 (0.86)
***Low Bulimia***		
*Valence*	4.8 (0.82)	5.5 (0.68)
*Arousal*	3.6 (1.25)	2.86 (1.44)
**High Drive for Thinness**		
*Valence*	4.0 (0.61)	5.3 (0.47)
*Arousal*	3.9 (0.91)	2.12 (0.94)
**Low Drive for Thinness**		
*Valence*	4.9 (0.72)	5.4 (0.64)
*Arousal*	3.38 (1.34)	2.75 (1.42)

Note: For valence and arousal, ratings ranged from 1 (extremely negative valence, extremely low arousal) to 9 (extremely positive valence, extremely high arousal).

Correlation analyses confirmed the relationship between valence and arousal ratings and ED symptoms. For body words correlations were found for the EDI subscales body dissatisfaction, but also for drive for thinness regarding valence (r = -.673, p<.001; r = -.487, p<.01) and arousal (r = .344, p<.05; r = .351, p<.05). Bulimia was significantly correlated with valence only (r = −448, p<.01). In addition, body words were also rated as significantly more negative in valence in women reporting high trait anxiety (r = -.396, p<.05). For neutral words, ratings were uncorrelated with ED symptoms, BMI or mood.

Memory data comprised only N = 30 subjects. Nevertheless, body related words were better remembered than neutral words in the unexpected free recall test, *word category*, *F*(1,29) = 289.4, p<.001. In contrast to ratings, memory effects did not differ significantly between low and high ED groups and effects were uncorrelated with EDI scales, depression, anxiety or body mass.

## Discussion

In the present study, startle, heart rate and self-report data were assessed during reading of body words and neutral words to investigate the motivational relevance of body schema representations in young women as a function of ED risk including drive for thinness, bulimia and body dissatisfaction.

Startle modulation did not differ during reading of body words and neutral words. Difference scores (contrasting for each individual startle amplitudes to body words relative to neutral words), however, showed a significant positive relationship with females’ self-reported body dissatisfaction on the EDI Inventory. In addition, ANOVAs confirmed greater startle amplitudes during reading of body words than during reading of neutral words in women reporting increased body dissatisfaction on the EDI.

During reading, information conveyed by a word is rapidly mapped onto previous experience triggering action tendencies of approach or avoidance if it is of strong emotional and motivational relevance for the individual. This has been shown in a number of previous studies investigating startle modulation in healthy individuals or individuals with personality disorder or psychosomatic complaints (pre-chronic pain) during confrontation with emotion words or disorder relevant material consisting of body- or pain-related adjectives [23], [24], [41], [42]. The present results provide further evidence for a link between symbolic processing and startle reflex modulation in women reporting body dissatisfaction and thus prominent negative thoughts and feelings about their own body. In women, negative thoughts and feelings about the body can give rise to negative self-schemata regarding the own body [20], facilitating priming of basal responses of defense during exposure to body relevant information.

Subjectively, body words were rated as more unpleasant and arousing by women high in body dissatisfaction, bulimia or drive for thinness, and valence and arousal ratings were also correlated with ED symptoms in general. Interestingly, body words were rated as generally lower in valence and higher in arousal and were also better remembered than neutral words in the whole female sample, suggesting a negativity bias in young women regarding evaluations of their own body irrespective of their risk for EDs, their affective state or current body weight. Feelings of being inappropriate, fat, and ugly, even though self-perception as overweight might be unjustified, are very common in young women [1], [2] and even higher in college students than in the normal population [43], [44].

Changes in heart rate differed significantly during reading of body words and neutral words, but in contrast to startle modulation HR effects were unrelated to ED symptoms. HR effects were also unrelated with BMI and interindividual differences in mood as was found for startle patterns. This suggests generally faster orienting responses towards body related concepts than neutral concepts in young women irrespective of their risk for EDs, body mass or related affective disorders. Previous research exploring reaction times to body- and food words in tasks such as the Stroop or the dot-probe task found stronger interference effects for body- and food related words compared to neutral words in females diagnosed with AN or BN compared to healthy controls, for reviews see [45], [46]. Nevertheless, it has been debated to what extent performance in these tasks reflects attentional biases towards food and body related information in EDs or systematic avoidance of such words [47].

According to cognitive models of EDs the development of cognitive schemata containing dysfunctional attitudes about eating and body appearance contributes to the etiology and maintenance of EDs [20]. Such maladaptive schemata serve to guide information processing in support of the inaccurate and harmful beliefs, thus contributing to the maintenance of EDs. Results of a more recent meta-analysis provide only partial support for this notion, although this may be due to the fact that only studies evaluating Stroop interference were included [48]. The findings of the present study, however, are in line with this suggestion. Whereas a general processing bias for body weight and shape related concepts might exist in young women regardless of ED related problems, priming of defensive responses was specifically related to eating disordered attitudes, body dissatisfaction in particular.

Many women enter college with subclinical levels of eating disorders, some developing the full picture of an ED only a few years later [44], [49]. Amongst the risk factors commonly considered, body dissatisfaction has been demonstrated to be the strongest predictor of body image disturbances in EDs among young women in a number of longitudinal studies [50]–[52]. The results of the present study show differences in motivational priming during processing of body and neutral words to occur in females with high body dissatisfaction.

Women who are dissatisfied with their bodies might have a higher risk for EDs because confrontation with a simple word appears enough to prime defensive maneuvers against potentially troubling information associated with one’s own body. On the other hand, in women not all information about the body is associated with increased body dissatisfaction. Several studies found that women report to be especially unhappy with particular parts of their body including mainly the lower parts of the body (stomach, hips, and thighs). This has led to the development of a number of scales such as the EDI asking specifically for dissatisfaction with these body parts. Most of the body words used in our study described the lower parts of the body and associated shape related concerns. This contrasts with many earlier ED studies using word stimuli. These have often used mixed lists (e.g. words pertaining to weight, shape, body parts or food) including adjectives reflecting general attitudes towards the body (e.g. fat, ugly, slim, etc.) possibly tapping into socially formed stereotypes of overweight (anti-fat bias), which are not specific to EDs [53]. Homogenous word lists describing only body parts or weight related concerns associated with these parts might provide a more adequate measure for the assessment of ED relevant body schemas by avoiding activation of general and thus ED unspecific body stereotypes.

### Limitations and Conclusion

The findings reported in the present study could have implications for future research and the prevention and treatment of EDs. Our results suggest that peripheral-physiological measures such as the startle reflex might be used as indicators of females’ risk for EDs in future studies. Even in a small sample such as the present one and with words as stimuli, motivational priming of defensive responses discriminated between females varying in self-reported ED symptoms. Future studies will be needed to clarify if the present results can be replicated in larger student samples with ED risk groups based on criteria other than median split (e.g. comparison of extreme groups) as designs based on median split groups have only limited explanatory power and should be treated with caution [54]. Regarding design and experimental-set up, control words of high arousal and negative valence could be included in further studies to determine if priming of defense reactions in women with risk for EDs is specific to body words. There are further issues that could be improved in future including the assessment of body mass (objective measurement versus calculation based on participants’ subjective self-report of weight and height).

Taking these limitations into account, it would be of theoretical and practical importance to find out how motivational priming in response to body words changes during the course and treatment of EDs. For instance, Spresser et al. [30] assessed self-report data and startle modulation during presentation of real, thin or thick own face pictures in college students. Higher levels of body dissatisfaction were associated with increased startle responses to the simulated high weight gain pictures. Friederich et al. [28] on the contrary did not find differences in startle modulation between anorectic and bulimic and healthy females during exposure to food pictures or pictures of slender models. It is well known that body image disturbances vary across the course of eating disorders, with often no signs of enhanced bodily emotional reactions such as heart rate or skin conductance being detectable in females with EDs during exposure to images of their own body [11], [55]. Given that over the course of EDs physical appearance changes (in terms of weight gain or loss), it would therefore be important to investigate if body schemata stored in memory change accordingly or whether they remain unaltered. Investigating changes during reading of body words across different levels of responding as done in the present study could be especially fruitful to this end.

## References

[1] Grabe S, Ward LM, Hyde JS (2008) The role of the media in body image concerns among women: a meta-analysis of experimental and correlational studies. Psychol Bull 134: 460–476. 18444705 10.1037/0033-2909.134.3.460

[2] Smeesters D, Mussweiler T, Mandel N (2010) The effects of thin and heavy media images on overweight and underweight consumers: Social comparison processes and behavioral implications. J Consum Res 36: 930–949.

[3] Sepulvea (2002) Body image alteration in eating disorders: a meta-analysis. Psychology in Spain 6: 83–95.

[4] Grogan S (2008) Body Image: Understanding body dissatisfaction in men, women and children (second edition). New York: Routledge.

[5] Hilbert A, Tuschen-Caffier B, Vögele C (2002) Effects of prolonged and repeated body image exposure in binge eating disorder. J Psychosom Res 52: 137–144. 11897232 10.1016/s0022-3999(01)00314-2

[6] Tuschen-Caffier B, Vögele C, Hilbert A, Bracht S (2003) Psychological responses to body shape exposure in patients with bulimia nervosa. Behav Res Ther 41: 573–586. 12711265 10.1016/s0005-7967(02)00030-x

[7] Gaudio S, Quattrocchi CC (2012) Neural basis of a multidimensional model of body image distortion in anorexia nervosa. Neurosci Biobehav Rev 79, 113–117. 10.1016/j.neubiorev.2012.05.003 22613629

[8] Beato-Fernandez L, Rodriguez-Cano T, Belmonte-Llario A, Martinez-Delgado C (2004) Risk factors for eating disorders in adolescents – a Spanish community-based longitudinal study. Eur Child Adolesc Psychiatry 13: 287–294. 15490276 10.1007/s00787-004-0407-x

[9] Seeger G, Braus DF, Ruf M, Goldberger U, Schmidt MH (2002) Body image distortion reveals amygdala activation in patients with anorexia nervosa - a functional magnetic resonance imaging study. Neurosci Lett 326: 25–28. 12052530 10.1016/s0304-3940(02)00312-9

[10] Uher R, Murphy T, Friederich HC, Dalgleish T, Brammer MJ, et al (2005) Functional neuroanatomy of body shape perception in healthy and eating-disordered women. Biol Psychiatry 58: 990–997. 16084858 10.1016/j.biopsych.2005.06.001

[11] Vocks S, Busch M, Grönemeyer D, Schulte D, Herpertz S, et al (2010) Neural correlates of viewing photographs of onés own body and another womeńs body in anorexia and bulimia nervosa: an fMRI study. J Psychiatry and Neurosci 35: 163–176. 20420767 10.1503/jpn.090048 PMC2861133

[12] Wagner A, Ruf M, Braus DF, Schmidt MH (2003) Neuronal activity changes and body image distortion in anorexia nervosa. Neuroreport 14: 2193–2197. 14625446 10.1097/00001756-200312020-00012

[13] Vögele C, Gibson L (2010) Mood, emotions and eating disorders. In Agras WS (Ed.), Oxford Handbook of Eating Disorders. Series: Oxford Library of Psychology: 180–205. Oxford University Press.

[14] Miyake Y, Okamoto Y, Onoda K, Shirao N, Okamoto Y, et al (2010) Neural processing of negative word stimuli concerning body image in patients with eating disorders: A fMRI study. Neuroimage 50: 1333–1339. 20045473 10.1016/j.neuroimage.2009.12.095

[15] Shirao N, Okamoto Y, Mantani T, Yamawaki S (2005) Gender differences in brain activity generated by unpleasant word stimuli concerning body image: an fMRI study. Br J Psychiatry 186: 48–53. 15630123 10.1192/bjp.186.1.48

[16] Dolcos F, LaBar KS, Cabeza R (2004) Interaction between the amygdala and the medial temporal lobe memory system predicts better memory for emotional events. Neuron 42: 855–863. 15182723 10.1016/s0896-6273(04)00289-2

[17] Shirao N, Okamoto Y, Okamoto Y, Otagaki Y, Morinobu S, et al (2003) Ratings of negative body image words, negative emotion words and neutral words by eating disorder patients and healthy subjects. Brain Sci Ment Disord 14: 141–147.

[18] Smeets MA, Ingleby JD, Hoek HW, Panhuysen GE (1999) Body size perception in anorexia nervosa: a signal detection approach. J Psychosom Res 46: 465–477. 10404481 10.1016/s0022-3999(99)00005-7

[19] Smeets MAM, Kosslyn SM (2001) Hemispheric differences in body image in anorexia nervosa. Int J Eat Disord 29: 409–416. 11285578 10.1002/eat.1037

[20] Vitousek KB, Hollon SD (1990) The investigation of schematic content and processing in eating disorders. Cognit Ther Res 14: 191–214.

[21] Lang PJ (1995) The emotion probe. Am Pychol 50: 372–385. 10.1037//0003-066x.50.5.372 7762889

[22] Lang PJ, Bradley MM, Cuthbert BN (1997) Motivated attention: Affect, activation, and action. In Lang, PJ, Simons RF, Balaban M (Eds.) Attention and Emotion: Sensory and Motivational Processes, Mahwah, New Jersey, Erlbaum: 97–135.

[23] Herbert C, Kissler J (2010) Motivational priming and processing interrupt: Startle reflex modulation during shallow and deep processing of emotional words. Int J Psychophysiol 76: 64–71. 20171998 10.1016/j.ijpsycho.2010.02.004

[24] Herbert C, Deutsch R, Sütterlin S, Kübler A, Pauli P (2011) Negation as a means for emotion regulation? Startle reflex modulation during processing of negated emotional words. Cogn Affect Behav Neurosci 11: 199–206. 21369874 10.3758/s13415-011-0026-1

[25] Sánchez-Navarro JP, Martínez-Selva JM, Román F (2006) Uncovering the relationship between defense and orienting in emotion: cardiac reactivity to unpleasant pictures. Int J Psychophysiol 61: 34–46. 16430981 10.1016/j.ijpsycho.2005.10.023

[26] Lang PJ, Bradley MM (2010) Emotion and the motivational brain. Biol Psychol 84: 437–450. 19879918 10.1016/j.biopsycho.2009.10.007 PMC3612949

[27] Vila J, Guerra P, Muñoz MA, Vico C, Viedma-del Jesús MI, et al (2007) Cardiac defense: from attention to action. Int J Psychophysiol 66: 169–182. 17706311 10.1016/j.ijpsycho.2007.07.004

[28] Friederich HC, Kumari V, Uher R, Riga M, Schmidt U, et al (2006) Differential motivational responses to food and pleasurable cues in anorexia and bulimia nervosa: a startle reflex paradigm. Psychol Med 36: 1327–1335. 16790080 10.1017/S0033291706008129

[29] Mauler BI, Hamm AO, Weike AI, Tuschen-Caffier B (2006) Affect regulation and food intake in bulimia nervosa: emotional responding to food cues after deprivation and subsequent eating. J Abnorm Psychol 115: 567–579. 16866597 10.1037/0021-843X.115.3.567

[30] Spresser CD, Keune KM, Filion DL, Lundgren JD (2012) Self-report and startle-based measures of emotional reactions to body image cues as predictors of Drive for Thinness and Body Dissatisfaction in female college students. Body Image 9: 298–301. 22305111 10.1016/j.bodyim.2011.12.005

[31] Watson D, Clark LA, Tellegen A (1988) Development and validation of brief measures of positive and negative affect: The PANAS Scales. J Pers Soc Psychol 47: 1063–1070. 10.1037//0022-3514.54.6.1063 3397865

[32] Hautzinger M, Bailer M, Worall H, Keller F (1994) Beck-Depressions-Inventar (BDI). Bern, Switzerland: Huber.

[33] Laux L, Glanzmann P, Schaffner P, Spielberger CD (1981) Das State-Trait-Angstinventar (STAI). Weinheim, Germany: Beltz.

[34] Garner DM, Olmsted MP, Bohr Y, Garfinkel PE (1982) The Eating Attitudes Test: Psychometric features and clinical correlates. Psychol Med 12: 871–878. 6961471 10.1017/s0033291700049163

[35] Garner DM, Olmstead M, Polivy J (1983) Development and validation of a multidimensional eating disorder inventory for anorexia nervosa and bulimia. Int J Eat Disord 2: 15–34.

[36] Bradley MM, Lang PJ (1994) Measuring Emotion: The Self-Assessment Manikin and the Semantic Differential. J Behav Ther and Exp Psychiatry 25: 49–59. 7962581 10.1016/0005-7916(94)90063-9

[37] Vó MLH, Conrad M, Kuchinke L, Urton K, Hofmann MJ, et al (2009) The Berlin Affective Word List Reloaded (BAWL-R). Behav Res Methods 41: 534–538. 19363195 10.3758/BRM.41.2.534

[38] Kissler J, Herbert C, Peyk P, Junghofer M (2007) Buzzwords: early cortical responses to emotional words during reading. Psychol Sci 18: 475–480. 17576257 10.1111/j.1467-9280.2007.01924.x

[39] Bradley MM, Codispoti M, Lang PJ (2006) A multi-process account of startle modulation during affective perception. Psychophysiology 43: 486–497. 16965611 10.1111/j.1469-8986.2006.00412.x

[40] Blumenthal TD, Cuthbert BN, Filion DL, Hackley S, Lipp OV, et al (2005) Committee report: Guidelines for human startle eyeblink electromyographic studies. Psychophysiology 42: 1–15. 15720576 10.1111/j.1469-8986.2005.00271.x

[41] Hazlett EA, Speiser LJ, Goodman M, Roy M, Carrizal M, et al (2007) Exaggerated affect-modulated startle during unpleasant stimuli in borderline personality disorder. Biol Psychiatry 62: 250–255. 17258691 10.1016/j.biopsych.2006.10.028

[42] Knost B, Flor H, Braun C, Birbaumer N (1997) Cerebral processing of words and the development of chronic pain. Psychophysiology 34: 474–481. 9260501 10.1111/j.1469-8986.1997.tb02392.x

[43] Johnson C, Connors ME (1987) Bulimia nervosa: A biopsychosocial perspective. New York: Basic Books.

[44] Kenardy J, Brown W, Vogt E (2001) Dieting and health in young Australian women. Eur Eat Disord Rev 9: 242–254.

[45] Dobson KS, Dozois DJA (2004) Attentional biases in eating disorders: A meta-analytic review of Stroop performance. Clin Psychol Rev 23: 1001–1022. 14729421 10.1016/j.cpr.2003.09.004

[46] Lee M, Shafran R (2004) Information processing biases in eating disorders. Clin Psychol Rev 24: 215–238. 15081517 10.1016/j.cpr.2003.10.004

[47] Engel SG, Robinson MD, Wonderlich SJ, Meier BP, Wonderlich SA, et al (2006) Does the avoidance of body and shape concerns reinforce eating disordered attitudes? Evidence from a manipulation study. Eat Behav 7: 368–374. 17056414 10.1016/j.eatbeh.2005.12.002

[48] Johansson L, Ghaderi A, Andersson G (2005) Stroop interference for food- and body-related words: a meta-analysis. Eat Behav 6: 271–281. 15854873 10.1016/j.eatbeh.2004.11.001

[49] Striegel-Moore RH (1993) Etiology of binge eating: A developmental perspective. In Fairburn CG, Wilson GT (Eds.), Binge eating: Nature, assessment, and treatment: 144–172. New York: Guilford Press.

[50] Attie I, Brooks-Gunn J (1989) The development of eating problems in adolescent girls: A longitudinal study. Dev Psychol 25: 70–79.

[51] Stice E, Ng J, Shaw H (2010) Risk factors and prodromal eating pathology. J Child Psychol Psychiatry 51: 518–525. 20074299 10.1111/j.1469-7610.2010.02212.x

[52] Stice E, Marti CN, Durant S (2011) Risk factors for onset of eating disorders: Evidence of multiple risk pathways from an 8-year prospective study. Behav Res Ther 10: 622–627. 10.1016/j.brat.2011.06.009 PMC4007152 21764035

[53] Brewis AA, Hruschka DJ, Wutich A (2011) Vulnerability to fat-stigma in women's everyday relationships. Soc Sci Med 73: 491–497. 21794968 10.1016/j.socscimed.2011.05.048

[54] Royston P, Altman DG, Sauerbrei W (2006) Dichotomizing continuous predictors in multiple regression: a bad idea. Stat Med 25: 127–141. 16217841 10.1002/sim.2331

[55] Vocks S, Legenbauer T, Wächter A, Wucherer M, Kosfelder J (2007) What happens in the course of body exposure? Emotional, cognitive, and physiological reactions to mirror confrontation in eating disorders. J Psychosom Res 62: 231–239. 17270582 10.1016/j.jpsychores.2006.08.007

